# Emotional experiences of elderly patients with Chronic Obstructive Pulmonary Disease using extended home oxygen therapy: A qualitative study on reports at a university specialized outpatient clinic in Brazil

**DOI:** 10.1192/j.eurpsy.2022.1210

**Published:** 2022-09-01

**Authors:** E. Turato, G. Bueno, L. Valadão, I. Paschoal, C.J. Campos, L.C. Martins

**Affiliations:** State University of Campinas, Laboratory Of Clinical-qualitative Research, Campinas, Brazil

**Keywords:** Chronic Obstructive Pulmonary Disease, Qualitative research, Long-term Home Oxygen Therapy, nursing care

## Abstract

**Introduction:**

What do patients talk during a clinic evaluation? What do they report besides referring physical complaints? It is crucial to value ’hidden’ symbolic issues under a conversation between patient and his/her doctors and nurses. Elderly people are at increased risk of developing Chronic Obstructive Pulmonary Disease (COPD) that requires the management of associated emotions. In advanced stages, they need to use Long-term Home Oxygen Therapy (LTOT) as part of treatment. Patients perceive difficulties with its use, generating anguish.

**Objectives:**

To explore meanings of emotional experiences as reported by patients regarding LTOT, seen in a public university outpatient service.

**Methods:**

Qualitative design. Semi-directed interviews with open-ended questions were carried out with seven elderly patients at Pulmonology Outpatient Clinic at General Hospital at University of Campinas, diagnosed with COPD and using LTOT in period 2019 to 2020. Data were analyzed using Content Analysis with the support of Webqda software. COREQ checklist was used.

**Results:**

Three categories emerged from interviews: (1) Changes of self-image perception with great dissatisfaction in not recognizing their selves physically. (2) Sadness with social isolation and feelings of awkwardness regarding themselves. (3) Affective aspects bringing the need to re-mean old family supports.

**Conclusions:**

Simply listening to reports of emotional complaints implies only a description of a clinical condition of the psychic sphere. Elderly patients with COPD bring psychological representations of their clinical condition that call for a symbolic interpretation. If such patients become aware of hidden meanings, they can better manage their fears and other uncomfortable feelings.

**Disclosure:**

No significant relationships.

